# Intestinal Obstruction

**Published:** 1876-06

**Authors:** R. G. Wharton


Intestinal Obstruction—*By R. G. Wharton, M.D.*—April 18, 1875, visited Mrs. McK., at 6 a.m. She had been suffering all night with violent pains in the abdomen, around the edge of the ribs, and in the right scapula. Mrs. M. is nearly sixty years of age, is very delicate, has had Bright's disease for several years. She ate a hearty dinner yesterday, was taken ill a few hours after, and vomited undigested food in the night. I found her very ill, restless, nauseated, and in great pain. Gave a hypodermic injection of one-fourth grain of morphia, with one-sixtieth grain of atropia, and ordered a dose of salts to be given as soon as the stomach should be quieted. She vomited the salts, but was relieved of pain for several hours. At 4 p.m. there was return of pain, some fulness of abdomen, nausea, pulse 125. Repeated the dose of opiate and atropia, and ordered another dose of salts.

19th. Found her very ill; vomited the salts ordered last evening; passed a very bad night, in great pain; abdomen tympanitic; no action of bowels; no escape of flatus; abdomen quite tender over caput coli; a tumor to be felt there. I diagnosed obstruction, most probably from impacted feces. Ordered fomentations to the abdomen; gave the morphia and and atropia as before, and ordered enemata to be administered every few hours. Evening—The enemata could not be retained, on account of the great swelling of abdomen, which was increasing; pulse 120; very restless and sore on least motion; to relieve the pain gave another opiate.

20th. The patient no better this morning; was tolerably easy from the opiate during the night, but, as its effects have passed away, complains very much; pulse 120; mouth dry; nausea; very weak; abdomen still larger; gave iced water to drink, but the distention was so great that she could take only one or two teaspoonfuls at a time. As the enemata could not be retained even one minute—in fact, they were forced back immediately—I tried the galvanic battery, in the hope of stimulating the peristaltic action of the bowels. Introduced a metallic bulb connected with the negative pole into the rectum, and applied the wet sponge of the positive over the tumor on the right side. Used fourteen cells, and for ten minutes, making second interruptions with the current changed. In a short time some flatus escaped under the flow of the current, and with very great relief; the pulse was reduced to 106; she felt much better. Two hours afterwards I used the battery again, with still more relief; escape of more flatulence. I then had an enema of iced water administered, which brought away some hardened feces, and with much flatulence. I now gave the hypodermic injection of one-fourth grain of morphia and one-fortieth grain of atropia, as she was beginning to get very restless. At 9 P.M. had another cold enema administered, which did not bring any fecal matter, only the water was a little stained. Gave another opiate.

21st. Passed a better night, and, as the effects of the opiate taken last night have passed off, she is beginning to suffer very much; cannot bear the least motion of body; complains very much of pain; pulse 110. Gave the hypodermic of morphia and atropia at 8 A.M., and ordered the iced enemata to be repeated several times during the day. They served to refresh her, to cool the bowels, and to quiet the nervous system. Evening—No action as yet; the injections come away a little colored; has taken a few teaspoonfuls of iced milk to-day; repeated the opiate at night, and cold enema afterwards.

22d. Passed tolerable night; complains of great weakness; pulse 105. The effect of last opiate has worn off, and she begins to suffer very much; repeated opiate and atropia, and ordered the administration of the enemata during the day, and to take iced milk. Passed some fecal matter twice during the day, with great relief; less soreness; less distention; another opiate at night.

23d. Found her very weak, but the abdomen less distended; pulse 115, and very weak; passed a little fecal matter in the night. Another opiate, to be followed by enema. Passed some fecal matter in the evening. Another opiate at night.

24th. Much better; less distention; less soreness of abdomen; pulse 98. Another opiate, to be followed by enema, which brought away quite a free evacuation; opiate at night.

25th. Still better; in fact, she is now convalescent; reduced the opiate to one-sixth grain of morphia; gave iced milk to drink, of which she took a good quantity, and from this time convalesced slowly, and in the course of a week was tolerably well again, but felt sore on the right side of the abdomen for several days longer.

*Remarks.*—The good effects of the of the battery were very evident in this case, and the first indication of any relief was from its use. Its tonic action, enabling the bowels to expel the flatulence which paralyzed their action, was plainly to be observed, and it was only after its use that the enemata could be retained. The iced water also afforded great relief, as there were signs of approaching inflammation in the impacted bowels.—*Phil. Med. Times.*

